# Whole‐exome sequencing identifies a novel mutation of *GPD1L* (R189X) associated with familial conduction disease and sudden death

**DOI:** 10.1111/jcmm.13409

**Published:** 2017-10-27

**Authors:** Hao Huang, Ya‐Qin Chen, Liang‐Liang Fan, Shuai Guo, Jing‐Jing Li, Jie‐Yuan Jin, Rong Xiang

**Affiliations:** ^1^ School of Life Sciences Central South University Changsha China; ^2^ Department of Cardiology the Second Xiangya Hospital of Central South University Changsha China

**Keywords:** GPDL1, nonsense, ventricular tachycardia, cardiac conduction diseases, whole‐exome sequencing

## Abstract

Cardiac conduction disease (CCD) is a serious disorder and the leading cause of mortality worldwide. It is characterized by arrhythmia, syncope or even sudden cardiac death caused by the dysfunction of cardiac voltage‐gated channel. Previous study has demonstrated that mutations in genes encoding voltage‐gated channel and related proteins were the crucial genetic lesion of CCD. In this study, we employed whole‐exome sequencing to explore the potential causative genes in a Chinese family with ventricular tachycardia and syncope. A novel nonsense mutation (c.565C>T/p.R189X) of *glycerol‐3‐phosphate dehydrogenase‐like* (*GPD1L*) was identified and co‐segregated with the affected family members. GPD1L is a crucial interacting protein of SCN5A, a gene encoded sodium channel α‐subunit Na_v_1.5 and mainly associated with Brugada syndrome (BrS). The novel mutation (c.565C>T/p.R189X) may result in a premature stop codon at position 189 in exon 4 of the *GPD1L* gene and lead to functional haploinsufficiency of GPD1L due to mRNA carrying this mutation will be degraded by nonsense‐mediated mRNA decay, which has been confirmed by Western blot in HEK293 cells transfected HIS‐GPD1L plasmid. The levels of GPD1L decreasing may disturb the function of Na_v_1.5 and induce arrhythmia and syncope in the end. In conclusion, our study not only further supported the important role of GPD1L in CCD, but also expanded the spectrum of *GPD1L* mutations and will contribute to the genetic diagnosis and counselling of families with CCD.

## Introduction

Cardiac conduction diseases (CCDs) are an inherited cardiac disease that may present as a primary electrical disease or be associated with structural heart disease 1. Diseases like long‐QT syndrome (LQTS), short‐QT syndrome (SQTS), BrS are relatively common among CCD with the clinical manifestations of arrhythmia, syncope or even sudden cardiac death (SCD) 2. Mutations in genes that encoding the cardiac voltage‐gated channels or their modify proteins are the main pathogeny of CCD. Among that, the cardiac sodium current is primarily conducted through the sodium channel protein Na_v_1.5 that encoded by *SCN5A* 3, 4. Meanwhile, GPD1L protein can also influence sodium current by modify the sodium channel. *GPD1L* was first described in 2002 5 and then identified as a new causative gene of BrS in 2007 6. Later, *GPD1L* mutations were also found in patients with sudden infant death (SID) 7. To date, only several mutations have been found in *GPD1L* that associated with CCD, mainly BrS.

*GPD1L*, the gene located at chromosome 3p24‐p22, encodes protein that contains a glycerol‐3‐phosphate dehydrogenase (NAD^+^) motif and shares 84% homology with glycerol 3‐phosphate dehydrogenase‐1 (GPD1) [8, 9].

In this study, we investigated a family with arrhythmia, dilated cardiomyopathy (DCM) and syncope. An obvious autosomal‐dominant inheritance has been observed in this family. We have employed whole‐exome sequencing (WES) in combination with arrhythmia‐related gene‐filtering to explore the possible causative gene for this family, a novel nonsense heterozygous mutation (c.565C>T/p.Arg189*) of *GPD1L* was identified may underlie the inherited arrhythmia. Western blot in HEK293 cells transfected HIS‐GPD1L plasmid further confirmed that the levels of *GPD1L* (c.565C>T/p.Arg189*) expression was degraded compared with the controls (Wt).

## Material and methods

### Subjects

This study was approved by the review board of the Second Xiangya Hospital of the Central South University. The proband and his relatives who participated in the study had been given written informed consent. After that, we investigated medical histories of all nine family members. Blood was collected from the affected proband and his family members. Subjects were examined by 12‐lead echocardiogram (ECG) and B‐mode ultrasound.

### Method

#### DNA extraction, exome sequencing and filtering

Genomic DNA was extracted by DNeasy Blood & Tissue Kit (Qiagen, Valencia, CA, USA). The main part of WES was performed in the Novogene Bioinformatics Institute (Beijing, China). The exomes were captured by means of Agilent SureSelect Human All Exon V6 kits, and the platform of high‐throughput sequencing was performed in Illumina HiSeq X‐10. Filtering strategies conformed to our previous study 10. And the genes list is provided in Table S1.

#### Mutation validation and co‐segregation analysis

Sanger sequencing was performed to confirm potential causative variants in the family. Segregation analysis was performed in all family members. Primer pairs used to amplify fragments encompassing individual variants were designed by Primer 5, and the sequences of PCR primers will be provided upon request.

#### *GPD1L* expression vectors

The wild‐type *GPD1L* cDNA with C‐terminal flag and His‐tag in the pEnter was designed by ourself. The R189X‐GPD1L nonsense mutation was engineered into the vector above using the TaKaRa MutanBEST Kit (Takara Bio, Otsu, Shiga, Japan).

#### Cell culture and transfection

The HEK293 cells were cultured in DMEM, high glucose (HyClone, Logan, UT, USA) supplemented with 10% foetal bovine serum (Biological Industries, Cromwell, CT, USA) at 37°C in 5% carbon dioxide. Cells were transiently transfected with either His‐GPD1L (WT) or His‐GPD1L (R189X) using Lipofectamine 2000 (Thermo Fisher Scientific, Waltham, MA, USA).

#### Western blot experiments

Protein samples were resolved on 4–12% Bis‐Tris NuPAGE gels, followed by standard Western blotting with anti‐His‐tag (Cell Signaling Technology, Danvers, MA, USA) and anti‐β‐actin (Cell Signaling Technology). Chemiluminescent signals were scanned, and integrated density values were calculated with a chemiluminescent imaging system (Alpha Innotech, San Leandro, CA, USA).

## Results

### Clinical feature

**Figure 1:**
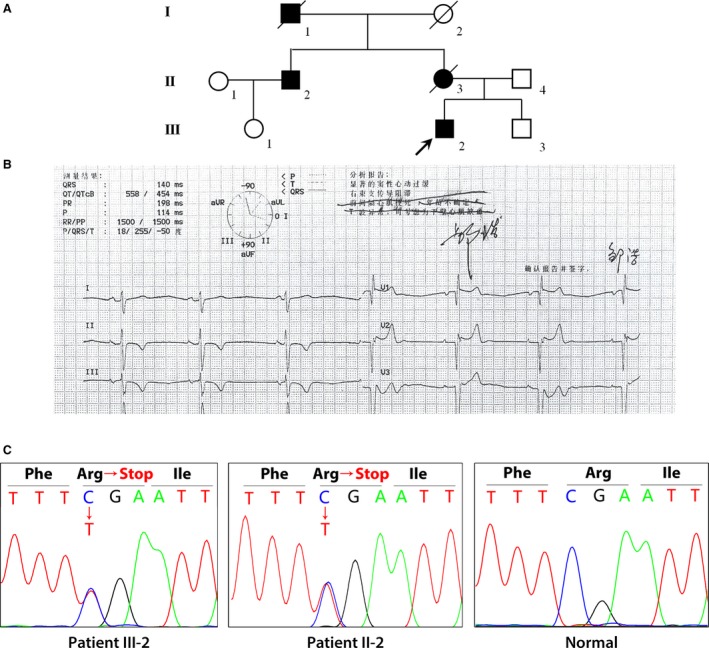
Clinical features of the patient with conduction diseases. (**A**) Symbols for affected individuals are coloured in. The proband (III‐2) was suffered from ventricular tachycardia, DCM and syncope. His mother (II‐3) and grandfather (I‐1) suddenly died with unknown reason. His uncle (II‐2) was also suffered from syncope for several times. (**B**) Electrocardiograms (ECGs) of the proband (III‐2). (**C**) Sanger DNA sequencing chromatogram demonstrates the heterozygosity for a GPD1L mutation (c.565C>T/p.Arg189*) in the proband and his uncle.

We described a Chinese family with multiple complex phenotypes including ventricular tachycardia and DCM (Fig. 1A, Table 1). The proband, a 20‐year‐old man, had an unprovoked palpitation from 2002 and diagnosed as left ventricular tachycardia in 2003, then discharged after radiofrequency ablation. During follow‐up, he still felt palpitation and attacked by syncope once in 2014. The ECG showed ventricular tachycardia (Fig. 1B). So he re‐hospitalized in 2014 for further treatment. The ultrasonic cardiogram revealed preserved biventricular enlargement (left atrial, LA 29 mm, left ventricle, LV 58 mm, right atrial, RA 32 mm, right ventricle, RV 36 mm) and LV systolic dysfunction (EF 48%). His mother and uncle were also suffered from syncope for several times. Additional, his mother and grandfather suddenly died during sleep for unknown reason.

**Table 1:** Summary of a family with ventricular tachycardia, syncope and sudden death

Family Member	Sex	Age (year)	ECG	Ultrasonic cardiogram	Comment
I1	Male	63 (Death age)	Unknown	Unknown	Sudden death with unknown reason
II_2_	Male	45	Ventricular tachycardia	–	Brief syncope
II_3_	Female	42 (Death age)	Unknown	Unknown	Sudden death with unknown reason
II_4_	Male	47	–	–	–
III_1_	Female	25	–	–	–
III_2_ (Proband)	Male	20	Ventricular tachycardia	Biventricular enlargement	Brief syncope
III_3_	Male	17	–	–	–

### Genetic analysis identified a novel segregating mutation in *GPD1L*

WES yielded 10.21 Gb data with 99.7% coverage of target region and 99.0% of target covered over 10×. After alignment and single nucleotide variant calling, 54,727 variants were identified in the proband. Approximate 374 single nucleotide variants and indels were picked out after filtering. Next, we used the arrhythmia‐related genes list to filter the rest variants, and a set of 10 variants in nine genes were identified. Bioinformatics analysis by MutationTaster, PolyPhen‐2 and SIFT also carried out (Table 2).

**Table 2:** Variants identified by WES in combination with arrhythmia‐related gene‐filtering in this family

Chr	POS	RB	AB	Gene Name	AA Change	MutationTaster	Polyphen‐2	SIFT
1	100330094	A	G	*AGL*	NM_000645:exon3:c.A562G:p.K188E	Disease causing (0.9999)	BENIGN (0.053)	Tolerated (0.62)
3	32188173	C	T	*GPD1L*	NM_015141:exon5:c.C565T:p.R189X	Disease causing (1)	–	–
4	114278984	G	A	*ANK2*	NM_001148:exon38:c.G9210A:p.M3070I	Polymorphism (0.7823)	PROBABLY DAMAGING (0.961)	Damaging (0.05)
6	76540265	A	G	*MYO6*	NM_001300899:exon5:c.391 + 3A>G	Disease causing (1)	–	–
6	152558082	G	A	*SYNE1*	NM_033071:exon108:c.C19856T:p.T6619M	Disease causing (0.9992)	BENIGN (0.161)	Tolerated (0.24)
7	91690697	G	A	*AKAP9*	NM_005751:exon23:c.G5725A:p.A1909T	Disease causing (0.9999)	PROBABLY DAMAGING (0.998)	Tolerated (0.00)
18	29101215	CTT	C	*DSG2*	–	Polymorphism (0.9999)	–	–
X	32404572	T	C	*DMD*	NM_004011:exon5:c.A506G :p.K169R	Disease causing (0.9876)	PROBABLY DAMAGING (0.994)	Tolerated (0.1)
1	116268178	GAAA	G	*CASQ2*	–	Disease causing (0.9999)	–	–
1	116268178	GAAAAA	G	*CASQ2*	–	Disease causing (0.9999)	–	–

CHR: Chromosome; POS: position; RB: reference sequence base; AB: alternative base identified.

Sanger sequencing indicated that only a novel nonsense heterozygous mutation (c.565C>T/p.Arg189*) of *GPD1L* co‐segregated with the affected family members (Fig. 1C). This novel mutation, resulting in a premature stop codon at position 189 in exon 5 of the *GPD1L* gene, was also not found in our 200 local control cohorts, dbSNP and Exome Variant Server database (http://evs.gs.washington.edu/EVS/).

### Functional characterization of mutant R189X GPD1L

**Figure 2:**
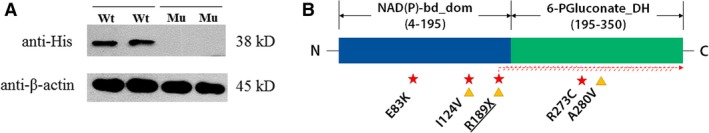
R189X nonsense mutation leads to functional haploinsufficiency of GPD1L. (**A**) HEK293 cells were transfected with either His‐GPD1L (WT) or His‐GPD1L (R189X), Western blot was used to detect the expression of His‐GPD1L and β‐actin. Lane 1 and 2, transfected HEK293 cells with human WT GPD1L; Lane 3 and 4, transfected with mutant R189X GPD1L. Wt, wild‐type; Mu, mutant. (**B**) Localization of the known mutations on the linear topology of GPD1L. Asterisks stand for mutations associated with sudden cardiac death. Triangles stand for the symptoms in those cases associated with Brugada syndrome. NAD(P)‐bd_dom, NAD(P)‐binding domain; 6‐P‐Gluconate_DH, 6‐phosphogluconate dehydrogenase C‐terminal domain‐like, Underline stands for present study.

The nonsense mutation of p.Arg189* was introduced into GPD1L cDNA clone as described in Methods. Mutant and wild‐type plasmids were transfected into the HEK293 cells, and Western Blot was used to examine the expression of mutant and wild‐type His‐GPD1L by anti‐His. R189X mutation showed no expression of His‐GPD1L in contrast to WT protein, whereas β‐tubulin level was unchanged (Fig. 2A).

## Discussion

In this study, we employed WES to explore the genetic lesion of a family with ventricular tachycardia, DCM, syncope and sudden death. A novel heterozygous nonsense mutation (c.565C>T/p.Arg189*) of *GPD1L* was identified and co‐segregated with the affected family members. This mutation was absent from the 1000 genomes, dbSNP144 and 200 local normal controls. Functional analysis revealed that this variant is deleterious. Western blot in HEK293 cells transfected HIS‐GPD1L plasmid further confirmed that the levels of *GPD1L* (R189X) expression was degraded compared with the controls (Wt). Based on the clinic phenotypes and genetic study, we can further make a definite diagnosis of BrS (a coved‐type ECG, syncope and family history of sudden death before 45 years old) to the proband and the affected family members.

In fact, just 16% of BrS patients can be explained by *SCN5A* mutation 11, with the other genes accounting for <5% of patients 12. As for *GPD1L* mutation, Makiyama *et al*. 13 have study 80 unrelated Japanese patients and screened for *GPD1L* mutations, no non‐synonymous mutations were found, and only one synonymous mutation and one intronic variant were identified, as well as a single nucleotide polymorphism detected in four patients. That seems the contribution of *GPD1L* mutations to BrS is probably rare in Japan. Winkel *et al*. 14 enrolled 66 non‐referred SCD patients born in Denmark and screened for genetic variants in the eight major genes including *GPD1L*. One rare variant of R220H (unknown significance) was found in an infant, who also carry another rare variant of *SCN5A*. In China, Liu *et al*. 15 were also found no non‐synonymous mutations in 123 medico‐legal autopsy‐negative sudden unexplained nocturnal death syndrome cases from Southern China. That seems the contribution of *GPD1L* to BrS is very rare and our study further provide a novel rare mutation of *GPD1L*.

To date, three mutations have been identified in *GPD1L* associated with BrS, including A280V, I124V and R189X (present study). Meanwhile three mutations, E83K, I124V and R273C were found associated with SID (Fig. 2B). Interestingly, the mutation I124V was reported twice to associate with SID and suspected BrS 16, respectively. Different from A280V, R189X nonsense mutation may trigger nonsense‐mediated mRNA decay (NMD) to prevent the production of truncated proteins 17, 18.

In conclusion, we report a novel rare *GPD1L* mutation (p.Arg189*) in a Chinese family with ventricular tachycardia, DCM, syncope and sudden death. Functional research indicated that this nonsense may lead to functional haploinsufficiency of GPD1L due to mRNA carrying this mutation degraded by nonsense‐mediated mRNA decay, which is confirmed by Western blot in HEK293 cells transfected HIS‐GPD1L plasmid. Our study not only further confirms the clinical diagnosis of BrS and provides a rare case, but also expands the spectrum of *GPD1L* mutations and may provide insight into genetic diagnosis and counselling of families with CCD and BrS.

## Conflict of interest

The authors declare that they have no conflict of interest.

## Supporting information


**Table S1** 103 cardiac conduction disorder‐genes for filter
