# Supplementary material for: Whole‐exome sequencing identifies a novel mutation of GPD1L (R189X) associated with familial conduction disease and sudden death
Source: J Cell Mol Med. 2017 Oct 27;22(2):1350–4. doi: 10.1111/jcmm.13409 (PMC5783853; doi:10.1111/jcmm.13409)
Supplement: Supplementary file 1 — Table S1 103 cardiac conduction disorder‐genes for filter [file JCMM-22-1350-s001.docx]

Supplementary Table S1. 103 cardiac conduction disorder-genes for filter

| *ABCC9* | *ACTN2* | *ACTC1* | *AGL* | *AKAP9* | *ANK2* | *ANKRD1* | *CAV3* | *CACNA1C* | *CACNB2B* |
| --- | --- | --- | --- | --- | --- | --- | --- | --- | --- |
| *CACNA2D1* | *CASQ2* | *CALM1* | *CALM2* | *CMD1B* | *CMD1K* | *COX15* | *CRP3* | *CRYAB* | *CSRP3* |
| *DES* | *DMD* | *DOLK* | *DSG2* | *DSP* | *DPP6* | *EMD* | *EYA4* | *FKRP* | *FXN* |
| *GBE* | *GLA* | *GPD1L* | *HCN4* | *HFE* | *HEY2* | *JPH2* | *JUP* | *KCNH2* | *KCNE1* |
| *KCNE2* | *KCNJ2* | *KCNQ1* | *KCNJ5* | *KCNJ8* | *KCNE5* | *KCND3* | *KCNE3* | *LAMP2* | *LCHAD* |
| *LMNA* | *MOG1* | *MYO6* | *MYOZ2* | *MYH6* | *MYH7* | *MYH11* | *MYBPC3* | *MYL2* | *MYL3* |
| *MYPN* | *PKP2* | *PLN* | *PRKAG2* | *RANGRF* | *RBM20* | *RYR2* | *RRAGC* | *SCN1B* | *SCN2B* |
| *SCN3B* | *SCN5A* | *SCN4B* | *SCN10A* | *SEMA4D* | *SGCB* | *SGCA* | *SGCD* | *SGCG* | *SLMAP* |
| *SNTA1* | *SYNE1* | *TAZ* | *TCAP* | *TGFβ3* | *T1EG1* | *TMEM43* | *TMPO* | *TNNI1* | *TNNT2* |
| *TNNI3* | *TNNI3K* | *TNNC1* | *TNNT2* | *TP63* | *TPM1* | *TTN* | *TTID* | *TTR* | *TRPM4* |
| *TRDN* | *VCL* | *ZASP* |  |  |  |  |  |  |  |
